# Mesoscale Fracture Analysis of Multiphase Cementitious Composites Using Peridynamics

**DOI:** 10.3390/ma10020162

**Published:** 2017-02-10

**Authors:** Amin Yaghoobi, Mi G. Chorzepa, S. Sonny Kim, Stephan A. Durham

**Affiliations:** College of Engineering, University of Georgia, Athens, GA 30602, USA; amin@uga.edu (A.Y.); kims@uga.edu (S.S.K.); sdurham@uga.edu (S.A.D.)

**Keywords:** multiphase concrete, mesh free, fracture analysis, state-based peridynamics, non-ordinary model

## Abstract

Concrete is a complex heterogeneous material, and thus, it is important to develop numerical modeling methods to enhance the prediction accuracy of the fracture mechanism. In this study, a two-dimensional mesoscale model is developed using a non-ordinary state-based peridynamic (NOSBPD) method. Fracture in a concrete cube specimen subjected to pure tension is studied. The presence of heterogeneous materials consisting of coarse aggregates, interfacial transition zones, air voids and cementitious matrix is characterized as particle points in a two-dimensional mesoscale model. Coarse aggregates and voids are generated using uniform probability distributions, while a statistical study is provided to comprise the effect of random distributions of constituent materials. In obtaining the steady-state response, an incremental and iterative solver is adopted for the dynamic relaxation method. Load-displacement curves and damage patterns are compared with available experimental and finite element analysis (FEA) results. Although the proposed model uses much simpler material damage models and discretization schemes, the load-displacement curves show no difference from the FEA results. Furthermore, no mesh refinement is necessary, as fracture is inherently characterized by bond breakages. Finally, a sensitivity study is conducted to understand the effect of aggregate volume fraction and porosity on the load capacity of the proposed mesoscale model.

## 1. Introduction

Concrete is the most widely-used construction material, and therefore, it is important to understand its fracture mechanism. Many studies consider concrete as a homogeneous material at the macroscopic scale. This assumption often results in inaccurate predictions of crack paths and load carrying capacity because concrete is a heterogeneous material. At the mesoscale, concrete is composed of four phases: coarse aggregate, air void, paste and the interfacial transition zone (ITZ), which exists between the coarse aggregate and paste. Mesoscale modeling is inevitable because the heterogeneity of concrete material is reflected at this scale and enhances the prediction of the fracture mechanism and macroscale behavior. As such, there has been increasing interest in mesoscopic material modeling to better characterize the fracture behavior in concrete.

Numerical simulations are an effective and practical alternative to experimental studies for investigating fracture in heterogeneous materials, such as concrete. Furthermore, analytical methods provide a useful tool for optimizing concrete mixtures by studying the effect of constituent material properties and variables, such as aggregate size, shape, surface texture, grading and volume fraction at the mesoscale. The use of imaging technology enables the determination of mesoscale particle size and location. The most recent imaging techniques include X-ray computed tomography scanning [1,2,3] to provide two- and three-dimensional mesoscopic aggregate and air void distributions.

### 1.1. Mesoscale Modeling

Mesoscale modeling using such imaging technology significantly enhances the accuracy of damage predictions. However, the application is not always practical. Two numerical alternative approaches exist utilizing a parameterization, which involves analytically generating heterogeneity: (1) by explicitly modeling different phases [4,5,6]; and (2) implicitly creating random fields, satisfying certain correlation functions [7,8,9]. In the direct explicit modeling approach, aggregates and air voids are randomly positioned within a specimen based on their sizes and gradation until the volumetric fraction is met.

The “take-and-place” method [4,5] has been widely used among several available techniques to numerically generate mesoscopic heterogeneous material phases. In this method, the coarse aggregate is randomly positioned within a domain. When an aggregate intersects with existing aggregates, the position is re-sampled. The maximum aggregate volume fraction that can be achieved by this method is approximately 60% [10]. Mier and van Vliet [11] developed the “random particle drop” method in order to achieve a higher aggregate volume fraction.

### 1.2. Analysis Methods Available for Fracture Modeling

Several models are available in the literature for fracture analysis of concrete members. Most popular models are based on the finite element method (FEM) [3,6,12,13,14,15], the discrete element method (DEM) [16,17] and lattice modeling methods [18,19,20,21,22,23]. The majority of fracture analysis models utilize the FEM, where for instance, failure is represented by inserting a zero-thickness cohesive interface element between homogeneous concrete elements. In this cohesive zone method (CZM), cohesive elements are surface elements that are placed along the element boundaries. Therefore, crack growth is mesh dependent because it occurs only between finite elements where cohesive elements are present. Furthermore, crack paths are highly sensitive to mesh alignment and/or refinement, particularly when they are unknown or unspecified a priori [3,6,13].

When strong discontinuities exist, advanced finite element methods, such as the extended finite element method (XFEM) [12,14], are used. The concept of XFEM was introduced as a technique that models crack initiation and growth within the realm of the FEM without mesh refinement. Local enrichment functions, with additional degrees of freedom, are included in the standard finite element approximation. Typically, the discontinuous displacement enrichment method is used to capture the displacement discontinuity across a crack and around a crack tip. Although XFEM has been successfully used to predict fracture in heterogeneous materials, an external criterion is needed to predict crack growth [12]. Major disadvantages of this method include significantly increased computational expense and complex enrichment functions, which make implementation difficult. Wavelet spectral finite element (WSFE) analysis and its implementation in Abaqus (WSFE-based UEL), developed by Khalili et al. [24,25], are among the novel FE methods in order to reduce the computational expense of finite element analyses.

In the discrete method, discrete elements (or particles) interact by means of contact forces. In the lattice modeling approach, a continuum is represented by a network of discrete elements. Schlangen and Van Mier [19] proposed a mesoscale model for simulating fracture in heterogeneous brittle materials utilizing the discrete modeling approach. Schlangen and Garboczi [21] studied the effect of lattice element type, mesh orientation and fracture criteria on the accuracy of fracture prediction.

While discrete and lattice modeling methods provide promising tools for fracture analysis of concrete at the mesoscale, characterizing parameters that define interactions among various phases in a heterogeneous material demands extra effort. Peridynamics is a non-local method first introduced by Silling [26]. The peridynamic method is well suited for modeling solid bodies with discontinuities. The solution algorithm is more robust than that of the finite element method because partial differential equations used in the classical solid mechanics are replaced by spatial integral equations (a sum of bond forces), which are also defined at discontinuities, such as cracks. Crack initiation and propagation are inherently represented by defining a bond relationship between particles using a constitutive model. Furthermore, the bond relationship between dissimilar material phases is inherently integrated in the peridynamic method.

Two methods are primarily used: bond-based peridynamics (BBPD) [26,27] and micro-polar peridynamics (MPPD) [28,29]. BBPD and MPPD assume that the bond force developed between material particle points is similar to those in a truss and a beam element, respectively. Therefore, these two methods resemble a lattice modeling approach. In addition to the similarity in defining the bond forces, peridynamics distinctly provide a set of material particles, whereas a continuum is discretized using a network of discrete elements in the lattice modeling method. Each material particle in peridynamics represents an infinitesimal volume and has its mass lumped at the center. The material continuity is provided by a “force-field” generated between particles.

In the bond-based peridynamics, independent interactions of a pair of material points were represented by a central-pairwise force. This assumption restricted BBPD to a Poisson’s ratio of 1/4 in three-dimensional models [27,30]. Although, MPPD addressed this issue by introducing rotational degrees of freedom into the bond-based peridynamic model [28], it was difficult to represent all aspects of material response, particularly when a collective response among material particle bonds was involved (e.g., a volume change). To avoid this type of restriction, a generalized peridynamic formulation, so-called non-ordinary state-based peridynamics (NOSBPD), was introduced. The main goal of NOSBPD was to allow interactions among the bonds [31]. Therefore, the collective deformation of the bonds around a material point defines the response of the point [32]. In NOSBPD, each bond between two material points is capable of carrying loads in all directions, which allows characterizing materials with any thermodynamically-admissible Poisson’s ratio [32,33,34]. Furthermore, the NOSBPD is capable of representing genuine material behaviors, such as a volume or shear angle change. Furthermore, the force state is represented by the classical stress and strain tensors, which enable the use of constitutive and damage models from the classical mechanics theory.

### 1.3. Summary of Work Presented in the Remaining Sections

This study includes a proposed mesoscale modeling approach in which heterogeneous concrete material is represented by four phases in the NOSBPD framework. A statistical approach is used to generate more realistic coarse aggregate size and distribution and to provide statistically-significant analysis results with a reasonable computational cost. Furthermore, crack or damage patterns are studied for varying particle spacing, aggregate volume fraction and air void content. Finally, the accuracy of the proposed model is compared with available experimental and analytical (or FEM) results.

### 1.4. Novel Aspects of the Research

The novel aspects of this study include, but are not limited to: (a) damage (e.g., crack initiation and propagation) inherently characterized by the peridynamic analysis framework; (b) promising potential for developing into a multiscale coupling approach with the FEM and, thus, for practical modeling applications; (c) a practical parametric design, including varying aggregate/air void distributions and material dense packing, available for material engineers; (d) enhancement in predicting the mesoscopic fracture mechanism in cementitious composites.

## 2. Proposed Mesoscale Concrete Model

In the majority of available studies, concrete is considered as a homogeneous material for simplicity. However, this assumption yields results that are inaccurate in predicting crack paths and load carrying capacity because concrete is a complex heterogeneous material. Its heterogeneity plays an important role in fracture analysis. Therefore, it is proposed that a two-dimensional mesoscopic model be used herein to account for the heterogeneity, whereas a microscopic model is feasible at the expense of additional computational resources in order to represent micro-structural inhomogeneity.

### 2.1. Mesoscopic Constituent Materials

Concrete is mainly composed of two phases at the mesoscale: coarse aggregate and paste. In addition, a third phase exists in the interface between the coarse aggregate and paste. Each of these three phases is heterogeneous in its composition. Proportions and characteristics of each phase vary with mixture composition. In the proposed mesoscale model, concrete is considered a heterogeneous material consisting of four phases to account for porosity: the coarse aggregate, air void, cementitious paste and the interfacial transition zone (ITZ), which exists between the coarse aggregates and cementitious matrix. In mesoscale modeling, the paste represents a mixture of microscale fine aggregates, cementitious hydration products, such as calcium-silicate-hydrate and calcium hydroxide, and water. Each phase is described below.

Cementitious matrix: A cementitious matrix itself at the microscale is a heterogeneous material that consists of fine aggregates, hardened cement paste with embedded pores. In the numerical mesoscale studies, cementitious matrix is assumed to be homogenous. The homogenized properties of the matrix are dependent on various factors, including cementitious type and content, water-cement ratio, compaction, which controls the amount of pores, and environmental conditions during the hardening process [35].

Coarse aggregate: Mechanical properties of concrete mixtures are highly affected by coarse aggregate material (limestone or granite), source, size, gradation, shape (river gravel or crushed gravel), texture and distribution. Most normal strength concrete mixtures are composed of 40%–50% coarse aggregates by volume and 60%–80% when combined with fine aggregates [36].

Interfacial transition zone (ITZ): The interfacial region is formed between the coarse aggregates and hydrated cement paste and yields the weakest link in cementitious composites due to higher concentrations of soluble calcium hydroxide in the region [35]. The properties of composites are often governed by the nature of this interface layer. This highly heterogeneous region with a thickness between 20 and 100 μm plays an essential role in the macroscopic behavior of concrete structures. Many experimental studies are devoted to characterizing the homogeneous mechanical properties of this ITZ. The results indicate that micro-cracks are mostly initiated from this region and that increased aggregate volume/size is associated with reduced tensile strength due to increased ITZ area [37,38].

Air void: Mesoscopic air voids are distributed throughout the cement paste and could lower the compressive strength of the concrete.

**Figure 1:**
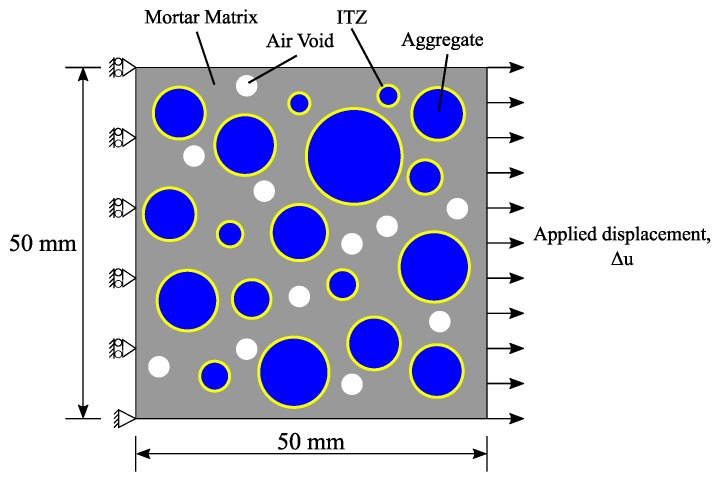
Schematic of mesoscale multiphase model subjected to tension.

**Figure 2:**
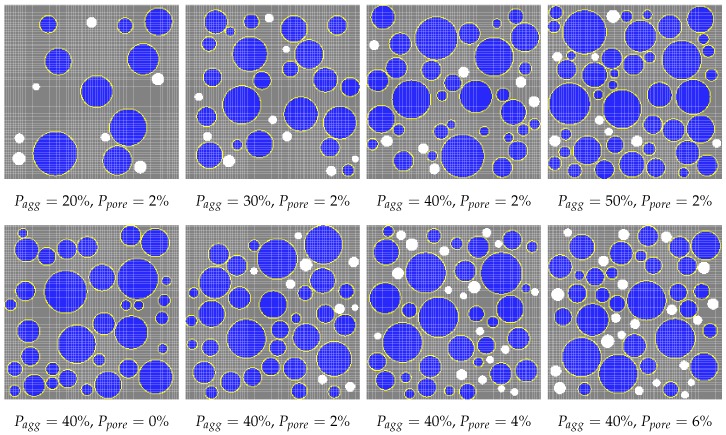
Numerical specimens with varying aggregate volume fraction and porosity.

**Figure 3:**
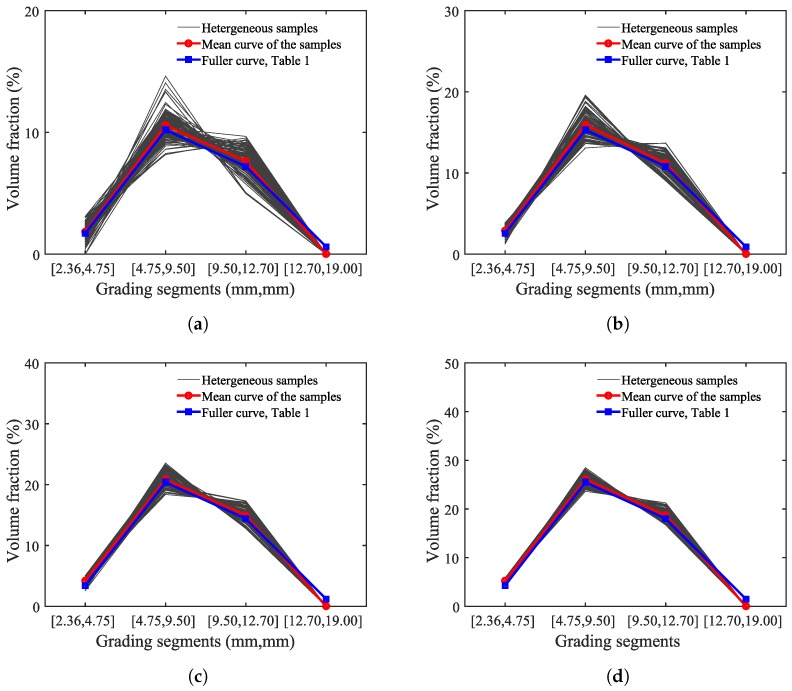
Aggregate gradations achieved by simulation versus the Fuller curve. (**a**) $P_{agg}=20\%$; (**b**) $P_{agg}=30\%$; (**c**) $P_{agg}=40\%$; (**d**) $P_{agg}=50\%$.

Figure 1 shows a schematic of four phases in a two-dimensional mesoscale concrete model. Figure 2 shows the position and distribution of the coarse aggregates and air voids in selected numerical specimens. In this figure, $P_{agg}$ and $P_{pore}$ represent the aggregate volume fraction and porosity, respectively. Figure 3 shows the gradation of the coarse aggregate for varying aggregate volume fractions described in the following section.

### 2.2. Distribution of Constituent Materials into Multi-Phases

Coarse aggregate: A random particle selection procedure is used in creating a mesoscale model. This particle placement approach, the so-called “take-and-place method” [4], is adopted in the proposed model to locate aggregates within an element domain. The aggregate shape is assumed circular, although other shapes such as angular are feasible and have been used in other mesoscale analyses [4,39]. Regardless, a grading curve must be provided to determine the volumetric ratio of the coarse aggregate and gradation. The Fuller curve is used in this study as it is widely employed to generate the optimum density and strength of concrete mixtures [19,36].

Equation (1) determines the cumulative percentage of aggregate passing a sieve with an aperture size of *d*, where $d_{max}$ is the maximum size of aggregate to be used, and the constant m=0.45−0.7 determines the shape of the grading curve. Equation (2) gives the total area of aggregates, $A_{agg}$, within the grading segment of $\left[d_{i},d_{i+1}\right]$, where $A_{model}$ is the total area of a numerical model and $d_{min}$ is the minimum aggregate size:

(1) $$P\left(d\right)=100{\left(\frac{d}{d_{max}}\right)}^{m}$$

(2) $$A_{agg}\left[d_{i},d_{i+1}\right]=\frac{P\left(d_{i+1}\right)-P\left(d_{i}\right)}{P\left(d_{max}\right)-P\left(d_{min}\right)}P_{agg}A_{model}$$

Beginning with the largest segment, the following procedure is used to determine the aggregate size and location in a given domain for each grading segment:

1. Determine the total area of the coarse aggregate, $A_{agg}$, with the aggregate size between $d_{i}$ and $d_{i+1}$, using Equation (1).
2. Generate a random number, which defines the aggregate diameter, *d*, within the segment $\left[d_{i},d_{i+1}\right]$. The aggregate diameter, *d*, is obtained by $d=d_{i}+\eta \left(d_{i+1}-d_{i}\right)$, where η is a variable selected from a uniform distribution of numbers between zero and one by using the “RANDOM_NUMBER” command in FORTRAN.
3. Generate two sets of random numbers to define the location of current aggregate. Two numbers are selected from a uniform distribution, with equal probability for all values, of the random variables between zero and one.
4. Check the placement of aggregate: Two conditions must be met to position the aggregate. First, the aggregate must be located within the analytical specimen boundary with a minimum clearance distance from the specimen boundary, $\gamma_{1}$. There must be no overlapping area between current aggregate and previously-placed aggregate, if any. A minimum distance of $\gamma_{2}$ between the two aggregates must be considered. These two conditions assure that the current aggregate is reasonably surrounded by the cementitious matrix. For the coarse aggregate distribution, 0.1d and $0.1(d+d^{\prime })/2$ are used for $\gamma_{1}$ and $\gamma_{2}$, respectively. *d* is the diameter of the current aggregate being positioned, and $d^{\prime }$ is the diameter of previously-positioned aggregate.
5. Repeat the random placement in Steps 2–3 until the conditions in Step 4 are satisfied.
6. Determine the total area of generated aggregates, $A_{agg}^{\prime }$, in this segment, and find the remaining aggregate area by subtracting $A_{agg}^{\prime }$ from the total aggregate area, $A_{agg}$, determined in Step 1.
7. Repeat Steps 2–6 until the remaining area is no longer available to generate additional aggregate in the grading segment.

Air voids: Once the generation and placement of the coarse aggregates is complete, the same procedure is used to create air voids ranging between 2 and 4 mm in diameter.

ITZ: A thin layer ranging between 20 and 100μm must be selected to represent the interface of coarse aggregate and paste. In this study, it is reasonable to select a single layer of particles surrounding coarse aggregates because the particle spacing studied herein is greater than 100 μm. With this simple assumption, ITZ and its associated damage is dependent on the particle spacing, although it is possible to use a smaller particle spacing in the ITZ. Therefore, it is essential to complete a convergence study to determine the particle size appropriate for characterizing the fracture mechanism in ITZ.

Cementitious matrix: The remaining particle points are used to form the cementitious matrix.

### 2.3. Formulation of the Proposed Analysis Framework

**Figure 4:**
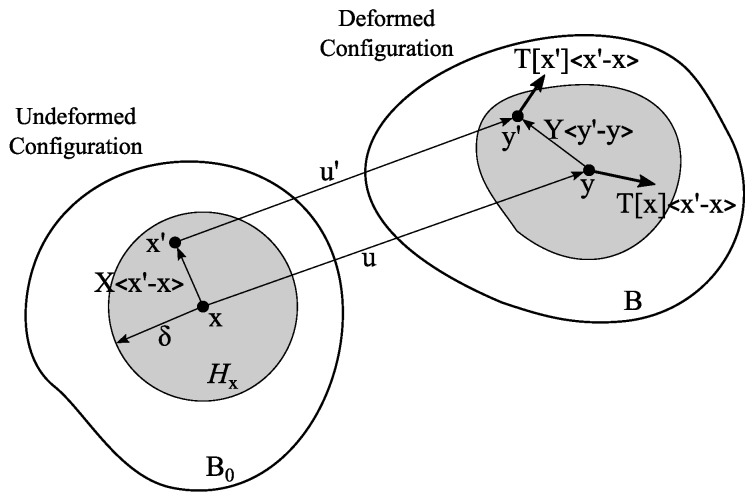
Kinematics of state-based peridynamics.

The kinematics of the peridynamics for a two-dimensional body is illustrated in Figure 4. A particle located at position x interacts with its surrounding particles within an area of influence, the so-called “horizon”, where δ is the horizon size. It is important to recognize the position vector-state, $X\langle x^{\prime }-x\rangle =\xi =x^{\prime }-x$. It is also referred to as the “bond” between two particles, x and $x^{\prime }$, and represents the relative position in the undeformed body, $B_{0}$. The deformation vector-state, $Y\langle x^{\prime }-x\rangle =\xi +\eta =y^{\prime }-y$, maps the bond, $X\langle x^{\prime }-x\rangle$, in the deformed body, B, where $\eta =u^{\prime }-u$ is the relative displacement of particles, x and $x^{\prime }$.

In the NOSBPD [32], the steady-state equilibrium equations for the particle, x, are given in Equation (3), where b is the body force applied on the particles, x, and $dV_{x^{\prime }}$ is the volume of the particle $x^{\prime }$. The force vector-state, T, is obtained by Equation (4), where $\omega \left(\xi \right)$ denotes a constant weight function and $\xi =\left|\xi \right|=\left|x^{\prime }-x\right|$. The stress tensor, σ, is the first Piola–Kirchhoff stress, and the shape factor, K, at particle x is defined by Equation (5). The symbol ⊗ denotes the tensor product.

(3) $$\int_{H_{x}}\left(T\left[x\right]\langle x^{\prime }-x\rangle -T\left[x^{\prime }\right]\langle x-x^{\prime }\rangle \right)dV_{x^{\prime }}+b\left(x\right)=0$$

(4) $$T\left[x\right]\langle x^{\prime }-x\rangle =\omega \left(\xi \right)\sigma \cdot K^{-1}\cdot \left(x^{\prime }-x\right)$$

(5) $$K\left(x\right)=\int_{H_{x}}\omega \left(\xi \right)\left[\left(x^{\prime }-x\right)⊗\left(x^{\prime }-x\right)\right]dV_{x^{\prime }}$$

A nonlocal approximation of the deformation gradient tensor, F, is defined by Silling et al. [32] to formulate the classical continuum mechanics’ constitutive equations in peridynamics:

(6) $$F\left(x\right)=\left[\int_{H_{x}}\omega \left(\xi \right)\left[\left(y^{\prime }-y\right)⊗\left(x^{\prime }-x\right)\right]dV_{x^{\prime }}\right]\cdot K^{-1}$$

The small strain tensor for isotropic elastic materials is determined by $\epsilon =1/2(F+F^{T})-I$, where the stress is obtained by σ=Cϵ; C is the isotropic elastic moduli matrix; and I is the identity matrix.

In the proposed approach, a higher-order polynomial approximation is used for the deformation gradient tensor in order to successfully suppress an instability problem found in NOSBPD [33,40]. Furthermore, an incremental and iterative solver is used to obtain the steady-state solutions. That is, the dynamic relaxation (DR) method is adopted to iteratively obtain solutions for a displacement increment, Δu. This iteration is necessary because the model oscillates about the equilibrium position until the size of the displacement vector for all particles becomes smaller than the dynamic relaxation threshold, ε [41,42]. This type of dynamic relaxation method determines steady-state solutions for a dynamic system by introducing fictitious mass and damping matrices and is particularly effective for solving highly nonlinear problems, including geometric and material nonlinearities.

There are several studies [33,43] that correlate the particle spacing, *h*, and horizon size, δ, to the rate of convergence in solutions. The vast majority of findings suggests that it is most effective when the horizon size is about three-times the particle spacing (i.e., δ≈3h) [44,45]. In this study, δ=3.1h is selected. Therefore, the particle spacing size, *h*, is the only variable that can affect the discretization size of a mesoscale model in this study.

### 2.4. Determination of Statistically-Significant Sample Size

One hundred samples are generated using the procedure described in Section 2.2 in order to study a statistically-significant numerical sample size. Two criteria are mainly used to determine the sample size: (1) average stress convergence and (2) average peak stress convergence.

## 3. Analysis of a Concrete Specimen in Tension

### 3.1. Description of a Numerical Model

A two-dimensional mesoscale representative model is studied to demonstrate the capabilities of the proposed method. Figure 1 shows a schematic of the numerical specimen and its loading condition. The specimen geometry and loading condition are selected because both experimental and analytical results are presented by Wang et al. [5], and thus, the analysis results can be verified by the cohesive FEM results and validated against experimental results. A quadrilateral discretization scheme is used to distribute material points needed for peridynamics analysis. The particle points generated within the air voids are removed from the analysis model as described in Section 2.2. Table 1 provides the gradation of the coarse aggregate obtained from Wang et al. [5].

### 3.2. Material Models

An isotropic damage model is used to describe the stress-strain relationships of constituent materials. Table 2 provides the material properties used for the analysis. The material softening due to micro-cracking is introduced in the material constitutive model using a damage variable, *D*, as shown in Equation (7):

(7) σ=(1−D)Cϵ

The damage variable, *D*, ranges from 0–1 for undamaged and fully-damaged materials, respectively, and is determined by Equation (8), where $\epsilon_{eq}$ is the equivalent strain determined by Equation (9), on the basis of the modified von Mises equivalent strain; $\epsilon_{0}=f_{t}/E$; $f_{t}$ is the tensile strength; $f_{c}$ is the compressive strength; $k=f_{c}/f_{t}$ and is assumed as 10. *E* is Young’s modulus; $\epsilon_{f}$ is the parameter affecting the slope of the softening branch; and $\epsilon_{f}=0.02$ is used in this study. This value is selected by comparing the analysis results obtained by the proposed peridynamic and finite element methods. That is, for each mesh size studied herein, a range of $\epsilon_{f}$ is considered for defining the softening curve. It is concluded that the mesh size has an insignificant effect on the parameter, $\epsilon_{f}$, when generating the PD analysis results that agree with the finite element analysis results. It is also concluded that the PD analysis results are insensitive for $\epsilon_{f}$ ranging between 0.019 and 0.021 and best agree with the FEA results when $\epsilon_{f}$ of 0.02 is used.

(8) $$D=\left\{\begin{array}{cc} 0 & :\epsilon_{eq}<\epsilon_{0}\\ 1-\frac{\epsilon_{0}}{\epsilon_{eq}}e^{-\frac{\epsilon_{eq}-\epsilon_{0}}{\epsilon_{f}-\epsilon_{0}}} & :\epsilon_{0}\leq \epsilon_{eq} \end{array}\right.$$

(9) $$\epsilon_{eq}=\frac{k-1}{2k\left(1-2\nu \right)}I_{1}+\frac{1}{2k}\sqrt{\frac{{\left(k-1\right)}^{2}}{{\left(1-2\nu \right)}^{2}}I_{1}^{2}+\frac{6k}{{\left(1+\nu \right)}^{2}}J_{2}}$$

In determining the equivalent strain in Equation (9), $I_{1}=tr\left(\epsilon \right)$ and $J_{2}=tr\left(\epsilon .\epsilon \right)-\frac{1}{3}tr^{2}\left(\epsilon \right)$ are the invariants of the strain tensor. The bond breakage procedure proposed by Tupec et al. [44] is used in this study. In their approach, a bond breakage criterion is applied to a pair of the particles, x and $x^{\prime }$. The weight function, used to define the bond between two points, x and $x^{\prime }$, is replaced with the following weight function:

(10) $$\hat{\omega }=\omega \left(\xi \right)\omega_{D}\left(D,D^{\prime }\right)$$

In Equation (10), $\omega \left(\xi \right)$ is the radially-symmetric weight function, which quantifies the reduced degree of interaction as a function of the distance away from the material points. $\omega_{D}\left(D,D^{\prime }\right)$ is zero if one of the particles is fully damaged; otherwise, it is one. *D* and $D^{\prime }$ is the damage parameter for x and $x^{\prime }$, respectively. Since Equation (8) does not yield one, the damage variable, *D*, is assumed as one (D=1) if *D* calculated by Equation (8) is greater than 0.99. This value is the critical damage threshold, $D_{cr}$, selected for this study.

In the peridynamic theory, the damage is associated with the bond breakage. In this study, the state of material damage is described by a single parameter, *D*, at each material point (see Equation (8)). Therefore, it is required to define a bond breakage criterion between a pair of particles, x and $x^{\prime }$, based on their damage parameters, *D* and $D^{\prime }$, respectively. Therefore, the damage parameters, *D* and $D^{\prime }$, are applied to compute the bond breakage threshold at two separate particle points, which belong to two material components, respectively. For example, the parameters are used to define the threshold force at the two particle points, which belong to a coarse aggregate and a cementations concrete matrix, respectively. It is important to note that the two damage parameters are also used to represent the damage between two particles from the same material (e.g., a coarse aggregate).

## 4. Analysis Results and Discussion of the Results

The analysis results indicate that the proposed mesoscale mesh-free approach combined with a simple material damage model is capable of characterizing the fracture behavior of the concrete specimen subjected to pure tension.

### 4.1. Effect of Particle Spacing and Convergence

**Figure 5:**
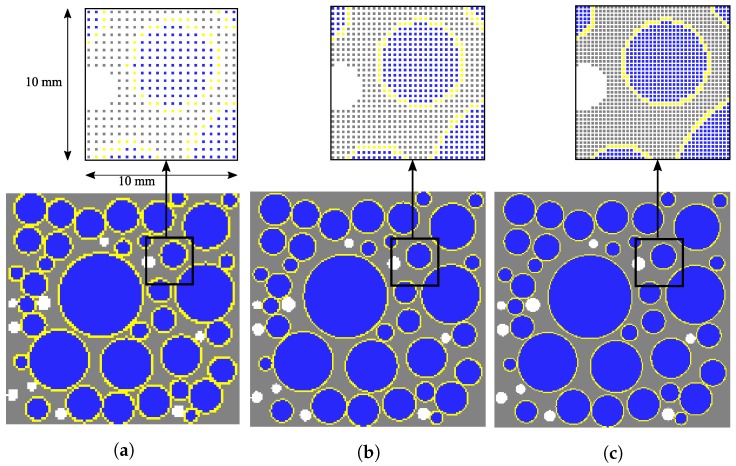
Numerical specimens with varying particle spacing, *h*. (**a**) h=1/2 mm; (**b**) h=1/3 mm; (**c**) h=1/4 mm.

**Figure 6:**
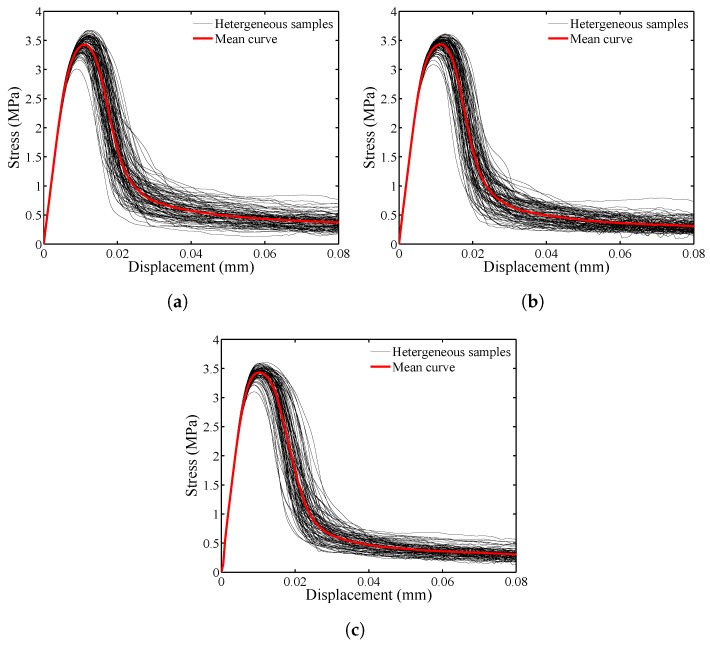
Tensile stress versus applied displacement for varying particle spacing. (**a**) h=1/2 mm; (**b**) h=1/3 mm; (**c**) h=1/4 mm.

In peridynamics, particle spacing and the horizon size are studied to determine the extent of discretization and convergence, whereas the mesh size is varied in the FEM. The specimen subjected to pure tension is discretized with three particle spacings, *h*: 1/2, 1/3 and 1/4 mm, as illustrated in Figure 5. For each discretization size, 100 heterogeneous models are sampled and analyzed, and the results are presented in Figure 6. The following solver parameters remain unchanged for this discretization study: $\Delta u=5\times 10^{-4}$ mm and $\varepsilon =10^{-7}$.

**Figure 7:**
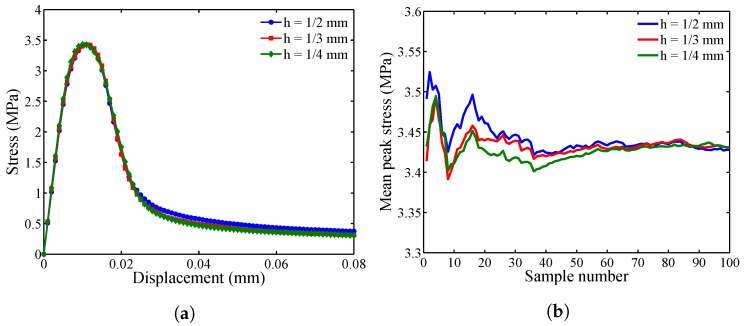
Effect of discretization on: (**a**) mean stress-displacement curves; and (**b**) sample number with reference to mean peak stress.

**Figure 8:**
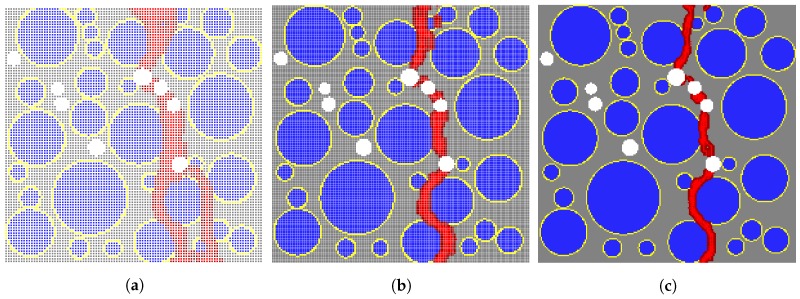
Effect of particle spacing size on crack pattern. (**a**) h=1/2 mm; (**b**) h=1/3 mm; (**c**) h=1/4 mm.

Figure 6 presents the applied displacement versus corresponding tensile stress computed from the mesoscale models for varying particle spacing, *h*. In the 100 heterogeneous samples, the stress-displacement relationship is highly nonlinear and always includes the linear-elastic and softening parts, regardless of the particle spacing size. However, the residual stresses deviate more from the mean curve for h=1/2 mm (see Figure 6a) than those determined when h=1/3 and 1/4 mm are used (see Figure 6b,c). Furthermore, it is clear from Figure 7a that the stress converges when the particle spacing of 1/3 mm or smaller is used. Figure 7b indicates that the mean peak stress converges when 70 or more samples are generated for the studied particle spacing. Figure 8 illustrates the crack pattern developed for each discretization scheme while aggregate location and position in the specimen remains unchanged. For h=1/2 mm, the damaged area in Figure 8a is more extensive than those observed in Figure 8b,c, which is anticipated because the horizon size increases as the particle spacing increases (δ=3h) in the proposed model. The damage patterns for h=1/3 and 1/4 mm are comparable.

In the aggregate placement procedure, a clearance distance from the specimen boundary is given to prevent cracks from initiating at the interface exposed to the surface. Therefore, in the results presented in Figure 8, the interface transition zone is not intended to be exposed to the bounding surface.

**Figure 9:**
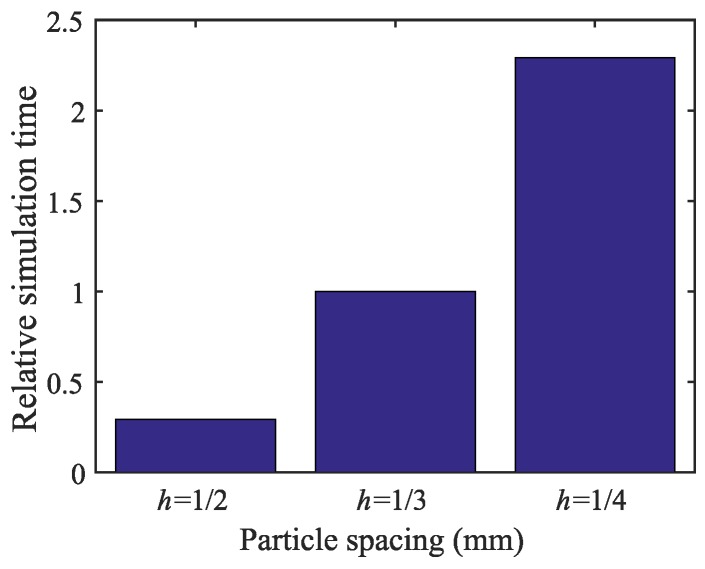
Effect of discretization on simulation time.

While the stress and damage pattern are effective indicators for convergence, the computational cost is another important factor in determining the particle spacing (or discretization size). Figure 9 shows the effect of particle size on the relative simulation time. It is scaled with respect to the simulation time needed for the specimen with h=1/3 mm. The analyses are completed using parallel computing and four processors on a UNIX cluster. The model with the particle size of 1/2 mm is four-times faster than that with h=1/3 mm, while the computational time decreases by a factor of 2.3 when the mesoscale model is discretized with h=1/3 mm, when compared to the model with h=1/4 mm. Therefore, based on stress convergence, damage patterns and the relative computational cost for the remaining study, mesoscale models are generated with the particle spacing of 1/3 mm and sample size of 100.

### 4.2. Effect of Loading Increment and Dynamic Relaxation Threshold

**Figure 10:**
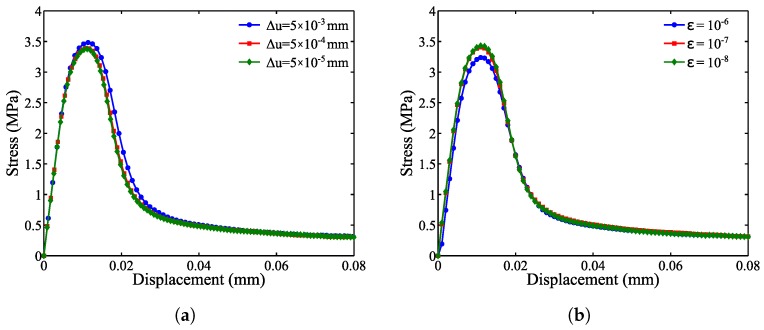
Effect of (**a**) the displacement loading increment and (**b**) the dynamic relaxation threshold on the mean stress curve.

To obtain the implicit solutions for a quasi-static peridynamic equilibrium equation (Equation (3)), an incremental and iterative method is developed. For a displacement state of δu, the dynamic relaxation method is adopted to iteratively update the displacement field, *u*, until $\left|\left|\delta u\right|\right|<\varepsilon$, where ε is a small numerical cut-off. Therefore, the steady-state solver parameters include the displacement loading increment, Δu, and dynamic relaxation threshold, ε. The effect of these two parameters on the accuracy and convergence of the analysis results is studied. Three displacement loading increments, Δu, of $5\times 10^{-3}$ mm, $5\times 10^{-4}$ mm, and $5\times 10^{-5}$ mm, are considered with a fixed value of $\varepsilon =10^{-7}$. As shown in Figure 10a, the stress results converge when Δu of $5\times 10^{-4}$ mm or smaller is used.

**Figure 11:**
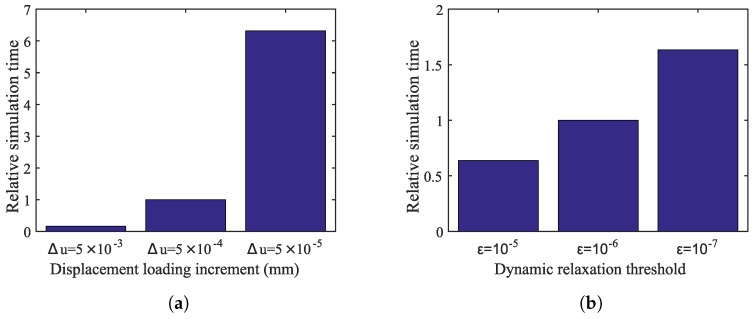
The effect of (**a**) the displacement loading increment and (**b**) the dynamic relaxation threshold on the simulation time.

The effect of the dynamic relaxation (DR) threshold, ε, on the analysis results is investigated. Three values of $10^{-6}$, $10^{-7}$ and $10^{-8}$ are considered for ε with a fixed $\Delta u=5\times 10^{-4}$ mm. The stress appears to converge when ε becomes $10^{-7}$ or smaller. Furthermore, the effect of these two variables on the relative simulation time is shown in Figure 11a,b, respectively. It is observed that the simulation time is highly (by a factor of six) affected by the change in the displacement loading increment, whereas the simulation time increases by 40%–50% when the dynamic relaxation threshold increases by a factor of ten.

### 4.3. Comparison with Available Finite Element Analysis Results

**Figure 12:**
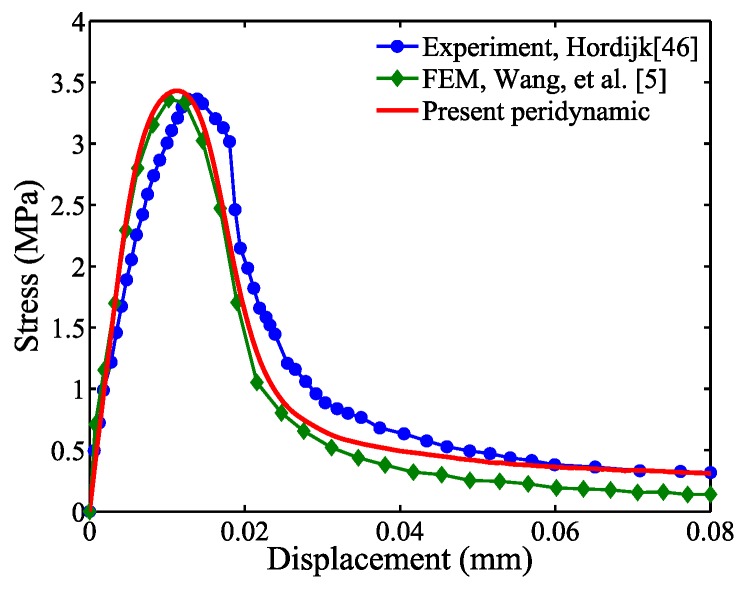
Comparison with experimental and available FEM results.

Based on the convergence study presented in Section 4.1 and Section 4.2, the mesoscale model is analyzed with the following two parameters: Δu of $5\times 10^{-4}$ mm and ε of $10^{-7}$, in conjunction with the particle spacing of 1/3 mm and sample size of 100. The analysis results are compared with the cohesive FEM results produced by Wang et al. [5] as shown in Figure 12.

Figure 12 indicates that the mesoscale model analyzed in the proposed peridynamics analysis framework yields similar results to the two-dimensional FEA results. This result is remarkable because Wang et al. [5] used multiple levels of mesh refinement and complex damage models to obtain the FEM results, whereas the proposed mesoscale model including four phases is relatively simple to generate, and the bonds between material particles are defined by simple damage models (see Section 3.2). The experimental results [46] are provided in Figure 12 as a point of reference and are discussed in Section 6.

## 5. Sensitivity Study

This section includes the sensitivity analysis capability of the proposed mesoscale model. Two variables, aggregate volume and porosity, are considered to study the effect of the variables on the stress results, although no experimental results are available to validate such analysis.

### 5.1. Effect of Aggregate Volume Fraction

**Figure 13:**
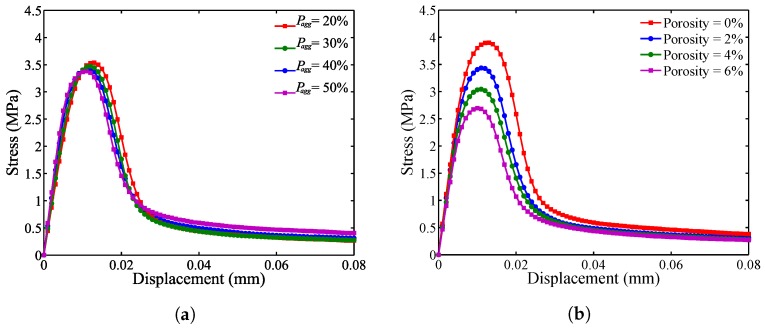
Effect of (**a**) aggregate volume fraction, $P_{agg}$, and (**b**) porosity, $P_{pore}$, on the mean stress curve.

Four coarse aggregate volume fractions, $P_{agg}$, of 20%, 30%, 40% and 50% are studied with a constant porosity, $P_{pore}=2\%$. For each of the four cases, 100 samples are studied to best represent the heterogeneous material phases. Figure 13a provides the stress-displacement relationship. The initial slope increases in the linear elastic region as the aggregate volume increases; however, the peak stress decreases due to increased ITZ resulting from increased aggregate volume, where the tensile strength is significantly lower than other materials. For the parameters studied herein, it is concluded that the peak load carrying capacity is reduced by increased aggregate volume. In general, the load carrying capacity may or may not increase [47,48,49], depending on the mix-design of cement paste and aggregate type selected.

### 5.2. Effect of Porosity

Porosity, $P_{pore}$, of 2%, 4% and 6% is considered to study the effect of air void content on the stress-displacement relationship. For each of the contents studied, 100 samples are analyzed with a constant coarse aggregate volume fraction, $P_{agg}=40\%$. Both the linear elastic modulus and peak load decrease as the porosity increases as shown in Figure 13b.

### 5.3. Comparison with the FEM Results and Damage Patterns

**Figure 14:**
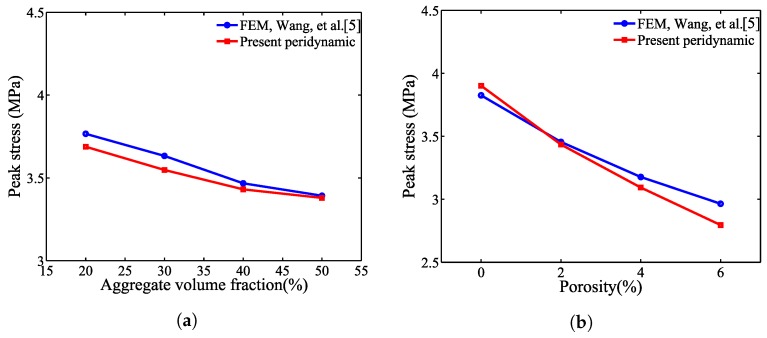
Mean peak stress for varying (**a**) aggregate volume fraction, $P_{agg}$, and (**b**) porosity, $P_{pore}$.

**Figure 15:**
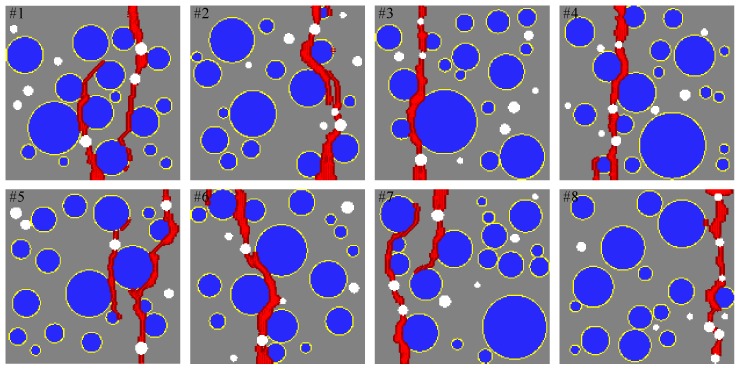
Crack pattern for selected specimens with $P_{pore}=2\%$ and $P_{agg}=20\%$.

**Figure 16:**
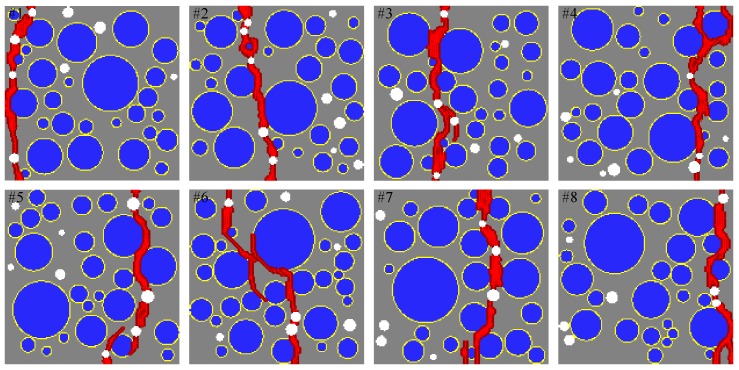
Crack pattern for selected specimens with $P_{pore}=2\%$ and $P_{agg}=30\%$.

**Figure 17:**
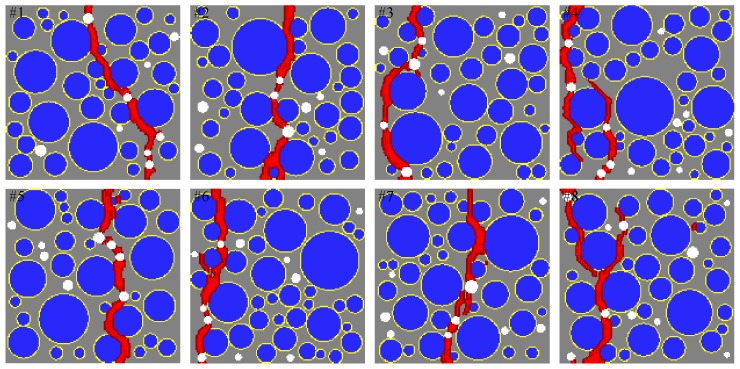
Crack pattern for selected specimens with $P_{pore}=2\%$ and $P_{agg}=40\%$.

**Figure 18:**
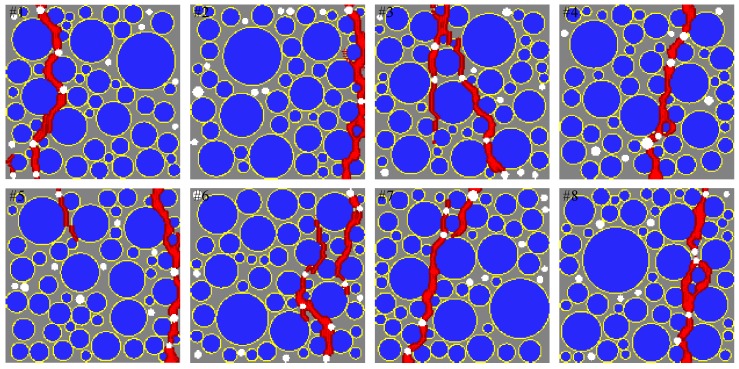
Crack pattern for selected specimens with $P_{pore}=2\%$ and $P_{agg}=50\%$.

**Figure 19:**
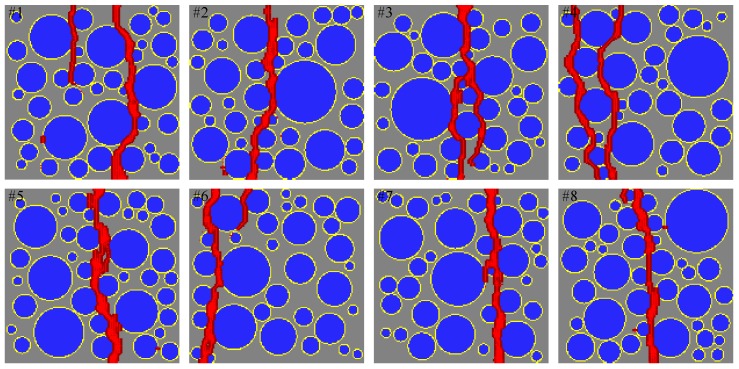
Crack pattern for selected specimens with $P_{pore}=0\%$ and $P_{agg}=40\%$.

**Figure 20:**
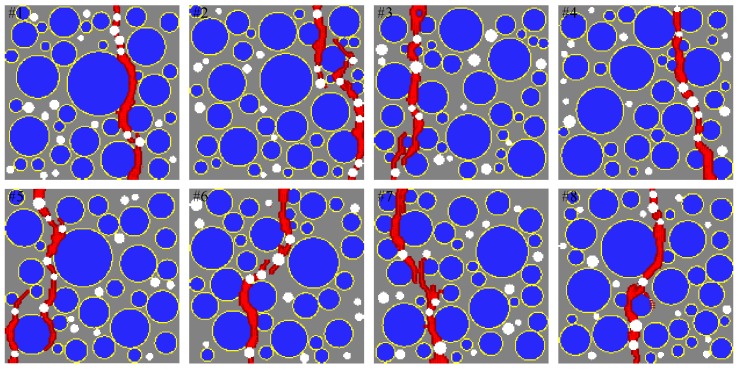
Crack pattern for selected specimens with $P_{pore}=4\%$ and $P_{agg}=40\%$.

**Figure 21:**
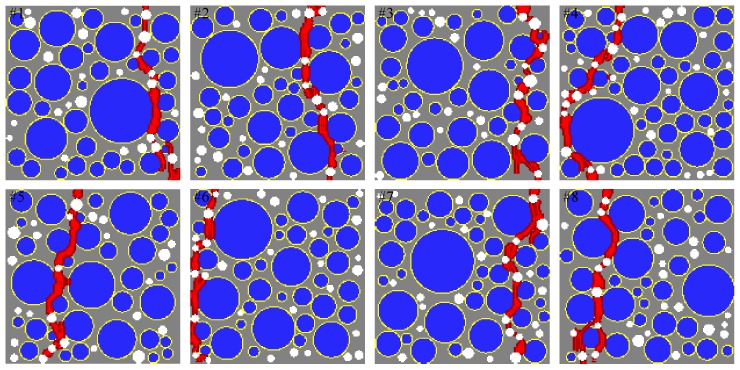
Crack pattern for selected specimens with $P_{pore}=6\%$ and $P_{agg}=40\%$.

Figure 14 shows a comparison of the sensitivity study results from the proposed mesoscale approach and FEM. The results are in good agreement despite the fact that considerably different analysis methods and material models are used in the mesoscale model. Figure 15, Figure 16, Figure 17, Figure 18, Figure 19, Figure 20 and Figure 21 present selected samples showing the crack patterns from the 100 samples used in the sensitive study. Seven selected cases corresponding to the four coarse aggregate volume fractions and four air contents are presented in the figures. It is concluded from this sensitivity study that the damage pattern is highly dependent on the distribution of the coarse aggregate and air voids and that one or two discrete cracks develop in the tensile specimen. It is also concluded from the proposed mesoscale model that the air voids and ITZs play an essential role in defining a crack path, which is not surprising, because they are the two weakest elements in the multiphase materials.

In Figure 15, Figure 16, Figure 17, Figure 18, Figure 19, Figure 20 and Figure 21, microscopic cracks, represented by damaged points in this model and thus invisible in these figures, originate from the interface zone. The weakest area involves increased numbers of air voids and interface zones. These micro-scale cracks coalesce, and the resulting mesoscale crack, which is visible in these figures, grows into the boundaries. As observed in the crack patterns, positioning an air void close to the surface does not always warrant a crack initiation.

## 6. Discussion

The stress-displacement curves agree well with available two-dimensional FEM results with the following limitations: (1) the numerical model includes the two-dimensional planar fracture assumption; (2) statistically-determined aggregate/air void distributions, which are physically different from the experimental specimen. Therefore, the experimental comparison only has qualitative meaning. The accuracy of the analysis is quantified by a comparison with the two-dimensional FEM results of Wang et al. [5]. It is also important to note that no damage is assumed to occur between the material points in an aggregate. This is attributed to a simple elastic material model used to represent the aggregate material. Therefore, in this model, cracks are not able to go through the aggregates because the elastic strength of aggregate is higher than that of the cement paste.

Mesoscale modeling of cementitious composites is challenging due to heterogeneous material constituents and the highly complex cracking mechanism. While the proposed approach builds a distinct framework for fracture analysis of concrete specimens, future work includes the implementation of the following features and tools for:

1. Studying the influence of particle shape and surface texture of the coarse aggregate.
2. Refinement in particle spacing and/or horizon size in ITZs to reduce the computational time while providing accuracy.
3. Enhancing material models and damaged parameters for complex loading conditions, such as shear.
4. Extending the proposed analysis approach to three-dimensional (3D) models in which, for instance, non-planar (or 3D) fracture surfaces are characterized.
5. Advanced computational techniques to optimize computational expense.

While the proposed mesoscale model is well suited to predict the fracture mechanism, comprising bond failure and crack initiation/propagation at the mesoscale, practical applications of this model may involve developing a successful coupling approach using both mesoscale and finite element modeling methods, in which concrete structures are practically characterized by finite elements. For the proposed approach to be pragmatic, the finite element model will need to be selectively refined using the multiscale coupling approach discussed in Section 1.4, which characterizes the mesoscale damage in a selected region.

## 7. Conclusions

The macroscale description of fracture behavior in cementitious composites is highly dependent on the heterogeneous constituent materials at a mesoscopic level. Therefore, it is important to characterize such a fracture mechanism in mesoscopic materials by using effective numerical techniques. In this study, it is found that mesoscale modeling of concrete members in a non-ordinary peridynamics analysis framework is highly effective for fracture analysis. The stress-displacement curves agree well with available experimental results and FEM results. The simple two-dimensional mesoscale model generated in peridynamics is well suited to understand the effect of gradation, aggregate distribution and proportions of constituent materials, as well as to predict the fracture mechanism, comprising bond failure and crack initiation/propagation at the mesoscale. The following conclusions are made by examining the results of this analytical study:

1. The results indicate that particle spacing affects the stress convergence, as well as crack patterns. For the tensile loading condition considered herein, the particle spacing of 1/3 mm provides the most effective discretization with a reasonable computational effort.
2. The results of the mesoscale analysis show that the simulation time is sensitive to the displacement loading increment used for dynamic relaxation. Based on the analysis results reported in this paper, the optimal displacement increment and DR threshold is $5\times 10^{-4}$ mm and $10^{-7}$, respectively, for tensile loading.
3. By means of interfacial transition zones (ITZs) characterized in the proposed mesoscale model, it is capable of reflecting the effect of varying coarse aggregate volume fractions on the load carrying capacity.
4. It is concluded that the strength reduction due to increased air content is reflected in the proposed model, by removing particle points from the areas of voids.
5. Finally, in the proposed mesoscale peridynamics analysis, it is possible to identify a statistically-significant sample size for reasonably representing coarse aggregate gradation and distribution and to predict the fracture mechanism in concrete specimens.

## Figures and Tables

**Table 1:** Gradation of the coarse aggregate [5].

Sieve size (mm)	19.00	12.70	9.50	4.75	2.36
Total percentage passing (%)	100	97	61	10	1.4

**Table 2:** Material properties [5]. ITZ, interfacial transition zone.

Constituent	Young’s Modulus, *E* (MPa)	Poisson’s Ratio, ν	Fracture Energy, $G_{f}$ (N/mm)
Aggregate	70,000	0.2	-
Mortar	25,000	0.2	0.06
ITZ	25,000	0.2	0.03
